# Adult Varicella Complicated by Deep Venous Thrombosis and Pulmonary Embolism: A Case Report and a Literature Review

**DOI:** 10.7759/cureus.59213

**Published:** 2024-04-28

**Authors:** Lizi Adishvili, Nino Bodokia, Sophio Tsikarishvili, Aleksandre Tskitishvili

**Affiliations:** 1 Medicine, AIETI Medical School, David Tvildiani Medical University, Tbilisi, GEO; 2 Cardiology, Caucasus Medical Centre, Tbilisi, GEO

**Keywords:** adult varicella, pulmonary embolism (pe), deep venous thrombosis (dvt), varicella zoster (chicken pox), s: chicken pox

## Abstract

Varicella-zoster virus (VZV) infection can rarely present with severe vascular pathologies, such as deep vein thrombosis (DVT) and pulmonary embolism (PE). These events are seldom documented in the literature as complications of primary VZV infection in adults. We present the case of a 52-year-old Caucasian male patient with chickenpox complicated by DVT and PE, which developed despite thrombectomy and anticoagulation. Laboratory analysis revealed elevated antiphospholipid antibodies. Although the patient was discharged home after clinical improvement, antiphospholipid antibodies remained elevated on repeat bloodwork eight weeks later. Our case report is followed by a literature review of 16 prior cases documenting primary VZV infection followed by DVT, PE, or both. The sex distribution of these cases, including ours, had a male-to-female ratio of 15:2. Mostly, DVT and PE occurred in the first and second weeks, underscoring the critical importance of screening for subtle thrombotic symptoms and risk factors for thrombosis during active VZV infection. Additionally, an argument can be made for ensuring the constant availability of the chickenpox vaccine for individuals with an increased baseline thrombotic risk, even if countries decide not to include varicella vaccination in their national immunization programs.

## Introduction

Varicella-zoster virus (VZV) is a DNA virus in the family Herpesviridae. While it primarily causes chickenpox, a self-limiting childhood illness, it can be much more dangerous in adults [1]. VZV can cause clinically significant disease during acute or recurrent infections, sometimes resulting in life-threatening complications. Cardiovascular complications of VZV infection are infrequent; a recent meta-analysis found a global pooled prevalence of only 0.55% (95% CI: 0.08-1.33) [2]. Out of these, antibody-mediated hypercoagulable states are mainly seen in children and are thus even rarer in adults [3]. Consequently, although deep vein thrombosis (DVT) following varicella was first described in the mid-1980s [4], the evidence linking primary VZV infection in adults to DVT and pulmonary embolism (PE) is still limited to case reports and a single small literature review of a neighboring topic [5].

We present a case of a VZV infection complicated by DVT and PE in an adult patient. We also introduce a literature review of 16 earlier cases, adding to and consolidating the evidence around the subject.

## Case presentation

A 52-year-old male patient presented to an ED in Tbilisi, Georgia, after being referred from a private clinic with a widespread vesiculopapular/pustular rash of eight days' duration. He also complained of fatigue, intermittent fever up to 39.5°C, myalgia, a sore throat, and a cough. For four days before the admission, the patient exhibited progressive bilateral lower extremity pain and swelling up to the knees, accompanied by petechiae and skin darkening. The systemic review was significant for a prior history of hypertension and unstable angina, which required coronary angiography and unsuccessful recanalization of multiple occluded arteries. He had been advised to take antihypertensive medications previously but had not been compliant with his regimen. The patient had a five-year smoking history, smoking one pack daily. Notably, he had never received any chickenpox or shingles vaccine.

On examination, he was febrile (38.5°C) and normotensive (118/78 mmHg). His respiratory rate was 19/min, heart rate 98 beats/min, and he maintained normal saturation on room air. He was alert and oriented to time, place, and person. The lesions on his skin were classic for chickenpox. Examination of the lower extremities revealed a swollen, tender, dark right calf with multiple petechiae. CNS, cardiac, chest, and abdominal examinations were all normal. Initial investigations showed tachycardia on an ECG and normal echocardiography results.

**Figure 1:**
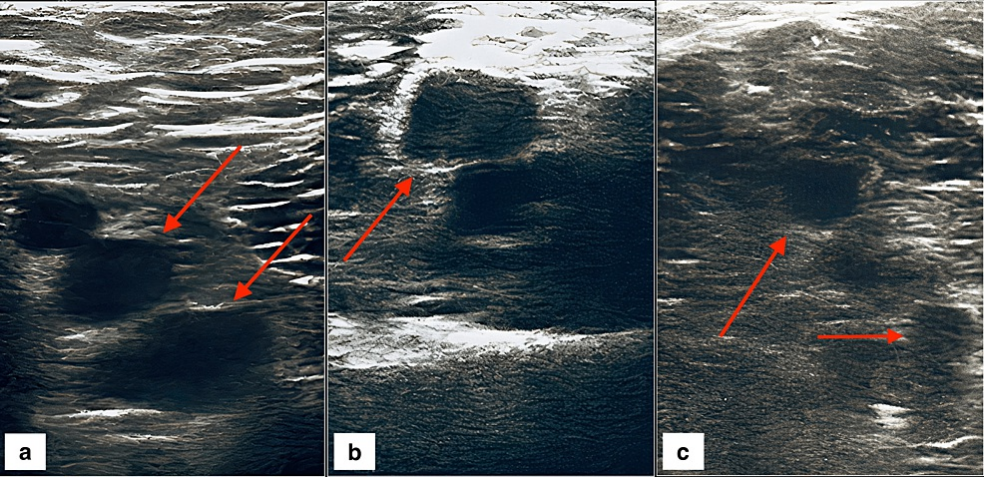
Thrombotic findings on ultrasound imaging upon the patient's admission. All images were taken during transducer compression of the veins. The inability to compress the vein indicates the presence of venous thrombi. Figure 1A shows non-compressible left femoral (upper arrow) and deep femoral (lower arrow) veins. Figure 1B shows the non-compressible right popliteal vein (arrow). Figure 1C shows non-compressible posterior tibial veins (arrow). Unlabeled lumens in all three images represent arteries.

Laboratory testing revealed elevated C-reactive protein, leukocytosis, decreased hemoglobin, elevated aPTT, and a normal INR. VZV serology using ELISA/ROIMMUN was ordered based on the patient's skin findings. A VZV IgM ratio of 2 (reference range: 0.8-1.1) and a VZV IgG ratio of 3.2 (reference range: 0.8-1.1) confirmed the active infection. Lower limb ultrasound (US) revealed bilateral DVT (Figures 1A-1C). Left saphenous vein thrombophlebitis was also found, necessitating thrombectomy and left crossectomy two days after the referral.

**Figure 2:**
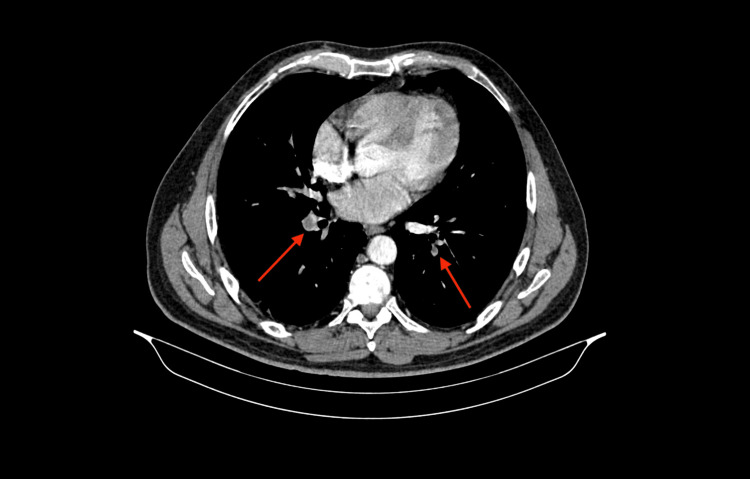
Bilateral pulmonary embolism after the surgery. Hypodense thromboembolic masses (arrows) on the thoracic CT angiogram.

After the surgery, the patient was transferred to the ICU and treated with subcutaneous heparin 5000 U every 12 hours. Over the next several days, the patient's condition improved. However, on the fifth postoperative day, petechiae reappeared, and he developed left femoral pain and swelling. A repeat US again confirmed DVT. Auscultation revealed diminished breath sounds over the lower zone of the right lung. Thoracic CT angiography showed multiple small hypodense thrombus masses in the lower segments of both pulmonary arteries, leading to a diagnosis of bilateral pulmonary thromboembolism (Figure 2).

Although the patient had no prior history of autoimmune or thrombotic conditions, the sudden, unexpected appearance of PE prompted laboratory tests to investigate the possibility of antiphospholipid syndrome. The patient spent several days in the ICU and was treated with 3.625 mg/day of oral warfarin for anticoagulation, along with acyclovir, aspirin, enalapril, and ivabradine. Vancomycin and piperacillin-tazobactam were added to prevent secondary infections. After about five days, the patient showed significant improvement, with a reduction in pain and swelling. He was discharged from the hospital but continues to be closely monitored and remains on warfarin, aspirin, and the previously mentioned antihypertensive medications. Repeat bloodwork done eight weeks after discharge confirmed the doctors’ clinical suspicions of antiphospholipid syndrome. Both in-hospital and follow-up laboratory test results are presented in Table 1. A repeat vascular Doppler US was recommended to be performed after six months.

**Table 1:** Laboratory analyses of antiphospholipid syndrome markers performed during hospitalization on the fifth post-surgical day and eight weeks after discharge.

Laboratory Test	Result During Hospitalization	Result Eight Weeks After	Reference Range
Anti-β_2_ glycoprotein I IgG	10.14 U/mL	10.09 U/mL	<7 U/mL
Anti-cardiolipin IgM	46.7 U/mL	48.3 U/mL	<44 U/mL
Antiphospholipid IgG	22.2 U/mL	25.78 U/mL	<15 U/mL
Antiphospholipid IgM	15.6 U/mL	16.91 U/mL	<15 U/mL
Lupus anticoagulant (LA) screen	45.6 seconds	44.08 seconds	31-44 seconds
LA confirm	42.5 seconds	Not performed	30-38 seconds

## Discussion

Although chickenpox is often regarded as merely a self-limited condition, its potential to cause uncommon but, at times, life-threatening complications has been well-documented. While more prevalent in children [3, 6], thrombotic complications of primary VZV infection in adults have also been increasingly recognized. In the last decade, cerebral venous sinus thrombosis (CVST) has received the most attention, especially in the context of VZV-related cerebral vasculopathy [7].

In contrast, evidence about DVT and PE as complications of VZV infection has only involved case reports so far. We performed a PubMed and Google search of literature in English, Spanish, French, German, Italian, and Russian, and Table 2 presents all the reported cases of adult primary varicella-associated DVT and PE we could find, including the one in our report. Cases related to herpes zoster were excluded.

**Table 2:** Seventeen cases of primary varicella infection in patients aged 18 and over (including the one in this report), complicated by deep venous thrombosis, pulmonary embolism, or both. aPL: Antiphospholipid antibodies; CVST: Cerebral venous sinus thrombosis; DVT: Deep venous thrombosis; LA: Lupus anticoagulant; PE: Pulmonary embolism; * : Poster presentation, not a case report. Note: Cases were found in English, Spanish, and French-language literature. Two case reports involving male patients were omitted due to being published in publications featured in Beall's list of possibly predatory journals.

Author	Age	Sex	Thrombotic Presentation	Laboratory Abnormalities	Days After the Start of Varicella Symptoms	Clinical Outcome
Ali MS et al., (1984) [4]	36	M	DVT	Not performed	5	Good recovery with anticoagulation, some residual swelling in the left leg
Gogos CA et al., (1993) [8]	36	M	DVT, PE	Normal	7 (DVT); +1 (PE)	Complete recovery of DVT with anticoagulation
Gogos CA et al., (1993) [8]	27	M	DVT	Not performed	5	Asymptomatic at discharge with anticoagulation
Huang DF et al., (1996) [9]	19	F	DVT, PE	Antinuclear and anti-dsDNA antibodies.	4 (DVT found, thrombi formed a few days prior); 8 (PE)	Death 20 minutes after acute massive pulmonary hemorrhage
Barcat D et al., (1998) [10]	42	M	DVT	aPL IgG positive on two samples 15 days apart; negative 45 days later. LA positive	2	Persistence of some pain on the 10^th^ day. Partial recanalization of femoral and iliac veins on MRI at four months. Anticoagulants for six months
Viseux V et al., (2000) [11]	29	M	PE	Normal	8	Asymptomatic at two months with anticoagulation. Mild interstitial changes on X-ray.
Rodríguez Borregán JC et al., (2003) [12]	33	F	DVT, PE	Not performed	Not specified	Good recovery on acyclovir. Anticoagulation status not mentioned
Rodríguez Borregán JC et al., (2003) [12]	40	M	DVT	Not performed	Not specified	Good recovery on acyclovir. Anticoagulation status not mentioned
Maldonado JA et al., (2004) [13]	27	M	DVT	aPL IgG	6	Favorable evolution upon treatment with acyclovir and anticoagulation
Dahan E et al., (2005) [1]	34	M	DVT, PE	Not performed	17 (DVT); +3 days (PE)	Clinical improvement with anticoagulation, no further episodes of hemoptysis
Paul G et al., (2016) [14]	37	M	CVST, DVT, and PE, a clot in the right atrium	Normal	Three weeks since the start (CVST), +5 days (PE); on acyclovir for the last 14 days	Gradual improvement with anticoagulation, discharged on day 12, no atrial clot on MRI after three months
Paul G et al., (2016) [14]	33	M	DVT	Normal	Two weeks	Gradual improvement with anticoagulation. Residual pain on prolonged walking at two months
Sandhya AS et al., (2018) [15]	20	M	DVT, PE	Not performed	5 (PE); +2 (DVT found on US)	Clinical improvement on anticoagulation, no further episodes of hemoptysis
Khan R et al., (2019) [3]	26	M	CVST, PE	Not performed	Two weeks (CVST), +2 days (PE)	Gradual improvement with anticoagulation
Gillispie A et al., (2020) [5]	52	M	DVT	Mildly elevated lupus anticoagulant	7	Gradual improvement with anticoagulation and valacyclovir. Discharged on day 7
Toujeni et al., (2023) [16] *	43	M	DVT	“No biologic sign of thrombophilia.”	15	Gradual improvement with anticoagulation
Adishvili L et al., (2023) (Current study)	52	M	DVT, PE	Elevated aPL IgM, IgG, anticardiolipin IgM, anti-β_2_ IgG and LA	4 (DVT); +7 (PE)	Gradual improvement with anticoagulation. aPL antibodies still positive after eight weeks

Notably, the sex distribution in these case reports is very lopsided. Out of the 17 cases presented in the table, only two were in females, resulting in a male-to-female ratio of 15:2. Even then, the female cases had powerful additional risk factors, such as systemic lupus erythematosus in one case and a history of smoking and oral contraceptive use in the other. Our statistics of VZV-associated DVT and PE somewhat resemble those of CVST. A literature review from 2021 found 13 CVST cases reported with a male-to-female ratio of 11:2, despite CVST caused by other factors being more common in females [17].

The literature review of VZV-related DVT and PE cases we undertook suggests that DVT and PE can develop anywhere from two days to three weeks after the onset of varicella symptoms. Therefore, it is essential not to let more dramatic presentations overshadow the often subtle thrombotic symptoms. For example, it may be helpful to study the risk-to-benefit ratio of assessing all hospitalized patients with chickenpox by lower extremity ultrasonography. This preliminary testing may be especially urgent in the presence of VZV's other thrombotic complications like CVST, since DVT and PE may sometimes develop in conjunction with other VZV-associated clotting states [13]. Out of these 17 cases, a single death occurred in a patient with a complicated lupus history. However, this sample size is not large enough to allow an appropriate comparison of the mortality rates between VZV-related and non-VZV-related thrombotic complications.

The pathogenesis of VZV-related thrombosis is multifactorial yet unclear and likely differs somewhat from case to case. In the past, various hypotheses have been put forward to explain these dangerous complications, including vascular inflammation caused by direct VZV wall invasion [14], autoantibodies causing local thrombosis [3], unmasking of hereditary thrombophilic states [3], direct endothelial damage, and infection-induced antiphospholipid syndrome [5, 11]. Interestingly, although transient deficiency of protein S due to autoantibodies is considered important in the pathogenesis of thrombotic complications of varicella in children [18] and has been discussed as a possible risk factor in adults [5, 11, 13, 15], no adult case of DVT or PE itself featured abnormalities of protein S (except for the one we decided to omit from publication included in Beall's list), in contrast to the cases with varicella complicated by CVST [14] or arterial thrombi [19]. This surprising lack indicates the need for further research.

Although our patient exhibited elevated levels of various antiphospholipid (aPL) antibodies, these can be present in as many as 20% of varicella patients [9], most of whom will not suffer any thrombotic complications. This high frequency of aPL, along with findings of aPL-negative thrombotic cases connected to anti-Protein S and anti-Protein C antibodies in children, has led to a hypothesis of a robust but non-specific antibody response to VZV infections [18]. Therefore, since the presence of aPL antibodies can be temporary and may not be pathogenic, the conclusion of their pathogenic role in a specific case should include the demonstration of the absence of other prothrombotic markers such as Factor V Leiden, and deficiencies in Protein S and Protein C. Khan also advises a prothrombotic screen after the patient’s recovery to diagnose hereditary prothrombotic states that require lifelong anticoagulants [3]. Additionally, it's essential to space the initial and repeat aPL tests at least 12 weeks apart, as the recently devised 2023 ACR/EULAR Antiphospholipid Syndrome Classification Criteria only consider positivity of both tests conducted at least 12 weeks apart as the criterion for persistent, rather than temporary, aPL antibody positivity [20].

The patient in our case report had never received either the chickenpox or shingles vaccine. Georgia's national immunization program does not include varicella vaccination, and its out-of-schedule availability is inconsistent. Consequently, the country witnessed a chickenpox outbreak in 2023, with more than 10,000 cases predicted within a year [21]. However, whether to include chickenpox vaccination in Georgia’s schedule is still highly debated. Officials argue that including chickenpox vaccination in the schedule may shift the age of infection to adulthood, when immunity wanes, thereby generating a higher risk of complications [22].

Therefore, it could be worthwhile to undertake further studies to identify or predict those at a higher risk of thrombotic complications (for example, studying males with a prior autoimmune history or polymorphisms predisposing to thrombophilia), and then, based on the evidence, argue for chickenpox vaccination and constant vaccine availability for these groups at least.

## Conclusions

DVT and PE are rare but potentially dangerous complications in adults with varicella, exhibiting an extreme sex bias towards males. These complications mainly arise in the first and second weeks, although they may sometimes occur later. We suggest further studying the risk-to-benefit ratio of lower extremity ultrasound screening in all hospitalized patients. Since the pathogenesis can vary from case to case and may involve various factors such as invasion-related, hereditary, or transient autoimmune phenomena, it may be prudent to assess a broad thrombophilia panel of antibodies and markers to determine their exact pathogenic roles and identify individuals with hereditary prothrombotic states who require lifelong anticoagulation. Further studies are needed to identify those at a higher risk of thrombotic complications to strengthen the argument for the constant availability of the vaccine in countries where chickenpox vaccination is not included in the mandatory governmental schedule.
